# Genomic Comparison of Indigenous African and Northern European Chickens Reveals Putative Mechanisms of Stress Tolerance Related to Environmental Selection Pressure

**DOI:** 10.1534/g3.117.041228

**Published:** 2017-03-22

**Authors:** Damarius S. Fleming, Steffen Weigend, Henner Simianer, Annett Weigend, Max Rothschild, Carl Schmidt, Chris Ashwell, Mike Persia, James Reecy, Susan J. Lamont

**Affiliations:** * Iowa State University, Ames, Iowa 50011; † Friedrich-Loeffler-Institut, 17493 Greifswald, Germany; ‡ University of Göttingen, 37073, Germany; § University of Delaware, Newark, Delaware 19716; ** North Carolina State University, Raleigh, North Carolina 27695; †† Virginia Tech University, Blacksburg, Virginia 24061

**Keywords:** tolerance, selective pressure, genomic variation, adaptation, environment

## Abstract

Global climate change is increasing the magnitude of environmental stressors, such as temperature, pathogens, and drought, that limit the survivability and sustainability of livestock production. Poultry production and its expansion is dependent upon robust animals that are able to cope with stressors in multiple environments. Understanding the genetic strategies that indigenous, noncommercial breeds have evolved to survive in their environment could help to elucidate molecular mechanisms underlying biological traits of environmental adaptation. We examined poultry from diverse breeds and climates of Africa and Northern Europe for selection signatures that have allowed them to adapt to their indigenous environments. Selection signatures were studied using a combination of population genomic methods that employed *F_ST_*, integrated haplotype score (iHS), and runs of homozygosity (ROH) procedures. All the analyses indicated differences in environment as a driver of selective pressure in both groups of populations. The analyses revealed unique differences in the genomic regions under selection pressure from the environment for each population. The African chickens showed stronger selection toward stress signaling and angiogenesis, while the Northern European chickens showed more selection pressure toward processes related to energy homeostasis. The results suggest that chromosomes 2 and 27 are the most diverged between populations and the most selected upon within the African (chromosome 27) and Northern European (chromosome 2) birds. Examination of the divergent populations has provided new insight into genes under possible selection related to tolerance of a population’s indigenous environment that may be baselines for examining the genomic contribution to tolerance adaptions.

The nature of specialized trait selection in livestock species within commercial operations, and in some instances experimental settings, has made them more sensitive to environmental extremes or rapid alterations in their environment (McMichael *et al.* 2007; Berry *et al.* 2011; Ciscar *et al.* 2011; van der Most *et al.* 2011; Kantanen *et al.* 2015). This issue may be a by-product of artificial selection within very controlled production environments (Thornton 2010; Herrero and Thornton 2013; Lawrence and Wall 2014) and is particularly true for genetically elite livestock and poultry. This susceptibility to environmental extremes is a hindrance to expansion of the poultry industry into areas of the world where the environment and its stressors drastically differ from the environment under which selection was performed (Canario *et al.* 2013; Lawrence and Wall 2014; Rothschild and Plastow 2014). Multiple environmental stressors triggered by climate change phenomena, such as extreme weather, can generate multiple stressors that test the ability of commercial livestock to survive and produce in harsh environments (Thornton *et al.* 2009; Ciscar *et al.* 2011). For livestock, environmental stressors can prove insurmountable when genetic potential and feed resource allocation are not matched (van der Most *et al.* 2011). Future agriculture will take place in more areas where changes in climate have made the environment less amenable to commercial livestock that lack the genetic potential to adapt (McMichael *et al.* 2007; Thornton *et al.* 2009; Rothschild and Plastow 2014). In particular, for poultry, an inability to cope with rapid or extreme changes to their environment can be costly and limits their potential in developing countries (Lara and Rostagno 2013; Porto-Neto *et al.* 2014; Rothschild and Plastow 2014) where poultry are vital for economic and nutritional importance (Neumann *et al.* 2002). To gain a better understanding of how to mitigate the impacts of environmental stressors by genetic approaches, it is prudent to examine native livestock species that have evolved under a given environment. For example, these could be landrace or village chickens indigenous to environments where environmental stress is endemic or mimicked (Seebacher 2009; Zakrzewska *et al.* 2011; Mwacharo *et al.* 2013; Porto-Neto *et al.* 2014).

The current study utilized populations of noncommercial indigenous African and Northern European (Tixier-Boichard *et al.* 2011) chickens to identify signatures of selection centered on survival of the environmental stressors of their respective habitats. The ability of the environment to apply selection pressure has been observed in studies of other livestock species. In sheep and swine, researchers were able to show correlations between selection signals and the local environment of different sheep breeds that were related to the shaping of adaptive variations (Lv *et al.* 2014; Ramirez *et al.* 2015; Yang *et al.* 2016). Given the extreme differences between the climate, geography, and the animals of Africa and Northern Europe, the selective pressure enforced by the environment should be detectable in the genomic architecture of the two deviating groups of populations (Hoffmann and Hercus 2000; Lyimo *et al.* 2014). By surviving in environmental conditions that can be considered as stressors for multiple generations, chickens indigenous to harsh environments should have been under selective pressure to develop tolerance at a genomic level (Clarke 2003; Benestad 2005; Chen *et al.* 2009; Seebacher 2009; Nardone *et al.* 2010; Lawrence and Wall 2014; Porto-Neto *et al.* 2014). Our study used this contrast of bird populations and high-density genomic variation data to examine how pressure from environmental stress can lead to advantageous genomic adaptions that allow for survival in harsh environments.

## Materials and Methods

The techniques used to analyze the population data allowed for the examination of selective pressure from the environment. The analysis workflow was composed of a combination of allele frequency and haplotype-based (Voight *et al.* 2006; Qanbari *et al.* 2011, 2012, 2014; Gautier and Vitalis 2012) detection methods performed on data generated with the 580k SNP Affymetrix Axiom Genome-Wide Chicken Genotyping Array (Kranis *et al.* 2013). These methods have previously been successfully employed to find areas under selective pressure that were associated with known and novel traits of biological interest (Voight *et al.* 2006; Qanbari *et al.* 2012, 2014; Gholami *et al.* 2014; Gutierrez-Gil *et al.* 2015; Kim *et al.* 2016). To account for the stochastic nature of environmental stress, temperature was used as a proxy to represent a dominant stressor that would have threatened survival without tolerance (Benestad 2005; Gerken *et al.* 2006; Seebacher 2009; Thornton *et al.* 2009; Walter and Seebacher 2009; Cheviron *et al.* 2014; Porto-Neto *et al.* 2014; Valero *et al.* 2014; Zhao *et al.* 2014). Temperature at extremes that confer stress across species has been well characterized by previous researchers (Gerken *et al.* 2006; Sharifi *et al.* 2010; Ciscar *et al.* 2011; Lara and Rostagno 2013; Mwacharo *et al.* 2013; Cheviron *et al.* 2014; Li *et al.* 2014; Lyimo *et al.* 2014; Porto-Neto *et al.* 2014; Valero *et al.* 2014; Zhao *et al.* 2014; Napper *et al.* 2015; Van Goor *et al.* 2015; Wang *et al.* 2015; Wu *et al.* 2015), who have shown it to affect the immune system, growth, and reproduction. This proxy, along with information from the National Oceanic and Atmospheric Administration (National Oceanic and Atmospheric Administration) on 30-yr average temperature and precipitation for each country, and the principal component analysis (PCA) based on the genotypic data from the array, were used to code the birds as “case” for higher ambient temperatures (African) and “control” for lower ambient temperatures (Northern European) for downstream analysis (Supplemental Material, Table S1).

### Animals

All animals (*N* = 718) were noncommercial indigenous breeds and ecotypes. All chickens except the Ugandan, Rwandan, and Kuroiler populations were provided by the Synbreed Chicken Diversity Panel (SCDP). The Ugandan, Rwandan, and Kuroiler data were taken from Fleming *et al.* (2016). The SCDP represents chicken breeds from: Africa (*N* = 375, Uganda, Rwanda, Kuroiler, Tanzania, Zimbabwe, Sudan, Egypt, Ethiopia, and Israel) and Northern Europe (*N* = 343, Germany, Iceland, Finland, Norway, and Poland). All birds were genotyped by using the Affymetrix 580k SNP chip and genomic DNA isolated from whole blood. Data of all birds sampled within a country were pooled to represent a region based on shared climate history (Table S1).

### Quality control

Plink 1.9 (Chang *et al.* 2015) was used to carry out quality control and filtering of the single nucleotide variants (SNVs) genotype data. Quality control for the samples allowed inclusion based on passing of the following filters: ≥0.05 for minor allele frequency (MAF), ≥99% individual call rate, and ≥99% SNV call rate. The genotyping rate was 0.998 for all samples. Eighty-six variants were removed because they mapped to the same chromosome and position. Only autosomal variants that passed QC were included in the downstream analysis. Filtering on the above parameters reduced the number of informative SNVs from 580,961 to 483,812 and the number of individuals from 718 to 634. The majority of the loss in sample numbers was the result of poor genotype calls for those samples that were removed during filtering. The 634 birds were portioned into 319 controls (European) and 315 cases (African) for downstream analysis of the remaining SNVs. The program Fastphase (Scheet and Stephens 2006) was used for haplotype phasing.

### PCA

The Plink 1.9 (Chang *et al.* 2015) PCA analysis module was used to identify covariate data to address the underlying subpopulations. The PCA was based on calculations of the variance-standardized relationship matrix of the genomic distances. The top three (*N* = 20) eigenvalues provided the clearest separation of the data and were used to construct the PCA plot.

### F_ST_ (case/control) analyses

Fixation test (*F_ST_*) analyses were conducted using Plink 1.9 (Purcell *et al.* 2007) to identify genomic regions of increasing genomic differentiation between the African and Northern European population samples. The populations were recoded into case/control categories using temperature as a proxy for environmental stressors. The case/control designation is based on a hypothesized natural selection for tolerance for higher ambient (African chickens) *vs.* lower ambient temperatures (Northern European chickens) as the case and control, respectively. Additionally, the *–family* argument was used in Plink 1.9 to account for possible stratification. Estimation of the genomic variation between the African and Northern European chicken populations was analyzed in Plink for *F_ST_*. Overlapping windows were generated using in-house Perl and R scripts. Windows were 100 kb and the step size was set to 50 kb to examine data with a 50% overlap The *F_ST_* results used for downstream analyses were based on the highest peaks (≥0.2). Peaks were measured by width of the base of the peak in order to determine regions for gene list creation. Differential allele frequency (AF) analysis was carried out by Plink to generate the MAF for each SNV by population and then used as a check of the *F_ST_* data.

### Selection signature analysis

Examination of the African and Northern European populations for signatures of selection was performed using the REHH package (Gautier and Vitalis 2012). Haplotypes were phased using FastPhase prior to signature of selection analysis. The REHH package was then used to calculate iHS to determine the existence of selection signatures within each population. Also, the standardized log-ratio of the integrated EHHS (iES) between pairs of populations (Rsb) was used for a pairwise examination of the populations for SNVs that displayed the population-specific selective pressure differences. Both iHS and Rsb values were log transformed to normalize the data and calculated following the method established by Voight *et al.* (2006). Statistical significance of iHS data was determined by first converting the −log p-values generated by the REHH software package back into nonlog p-values. From here, a permutation test was applied to the nominal p-values using the R package Perm (Fay and Shaw 2010) to shuffle p-values and perform a permutation *t*-test to set the threshold for significance, as a means of setting the adjusted p-value without the use of multiple test corrections. The threshold for statistical significance was set using permutations based on using both 1000 and 5000 iterations to set the adjusted p-value. Based on the permutation test, the corrected p-value was set at ≤ 3.8 × 10^−4^. The adjusted p-value was then reduced to ≤ 1 × 10^−4^ as an additional stringency to help account for using temperature as a proxy phenotype for the case/control-based comparisons. The reduced p-value (≤ 1 × 10^−4^) corresponded to an iHS score ≥ 3.89 for a variant to be considered as showing evidence of selection. This iHS value was used for both populations as the cut-off for variants, and the genes annotated to them that were later used for downstream gene ontology analysis (GO). The nature of the iHS test is based on a normalization of data making it possible to view iHS scores as deviations from the mean (iHS normalized mean = 0) represented by extremes values outside three SDs. Statistically significant iHS variants that cluster within intragenic regions indicate a gene considered to be under strong selection. Other variants in the African and Northern European chickens considered as being under strong selective pressure and possible candidate genes were annotated within 2 Mb upstream or downstream of statistically significant variants. Statistical significance for the Rsb analysis was set at a Rsb value of ≥4 and an adjusted p-value ≤ 1 × 10^−4^. Gene lists for downstream analysis were compiled in the same manner as for the iHS data. Candidate genes were selected based upon variant clustering within regions.

### ROH analyses

ROH are areas of a genome that harbor long stretches of homozygous genotypes. These stretches represent regions of low heterozygosity and could signify genomic hitchhiking related to signatures of selection (Lencz *et al.* 2007). The ROH analyses were carried out in Plink 1.9 to examine the populations for the presence of unique ROH. ROH were calculated using a sliding window approach and defined by the following parameters within Plink: SNVs ≥ 100, length ≥1 kb, ≤3 heterozygous calls to account for the presence of genotyping errors and/or hitch-hiking events, and window-threshold ≤0.05; no missing calls were allowed and program defaults were used for all other parameters. Overlapping ROH were considered matching based on pair-wise allelic matching of 95% or greater concordance. The analysis used sliding windows to determine the genomic regions of overlap within each population (African/Northern European). Only the overlapping consensus ROH regions, as defined by Plink 1.9, of the ROH were used in downstream GO analysis. The ROH analysis included the 30-yr mean temperature data in the form of the case/control designations that were based on historical high and low ambient temperatures (National Oceanic and Atmospheric Administration), respectively, to represent the environment or “landscape” indigenous to each population grouping (African/Northern European). Regions were considered unique if the consensus regions belonged to only one population (African/Northern European). All consensus ROH matching the following criteria were labeled as “landscape ROH” (LROH): (a) ≥1 kb in length; (b) the region was consensus among all members (chickens) of a “pool” (*i.e.*, overlapping ROH segments); (c) members of the pool represented ≥ ∼5% of the total number of samples assigned to that temperature zone (low ambient/Northern European or High ambient/African); and (d) contained only birds from one temperature zone. Gene lists used for downstream analysis were based on genes located between the first SNV of the consensus ROH and the last SNV of the last consensus ROH of that chromosome. Analysis of overrepresented GO terms for functions, processes, and pathways were based on a 5 Mb window based on a start position of −2 Mb from the first SNV of the LROH and +2 Mb from the last SNV in the LROH.

### GO enrichment analyses

Lists were created from genes annotated to statistically significant variants from each analysis and examined for overenriched processes and functions. The analysis approach used was termed “(w)HOL(e)ISTIC GO enrichment” and centered on the overlap of terms showing enrichment from multiple methods implemented in different software programs. The programs used consisted of GOtermfinder (Boyle *et al.* 2004), STITCH 4.0, STITCH 5.0 (both used due to version 5.0 being an update still in β) (Kuhn *et al.* 2014), DAVID (Huang *et al.* 2009; Jiao *et al.* 2012), g:Profiler (Reimand *et al.* 2011), and the Panther online database (accessed January 2016) (Ashburner *et al.* 2000; Gene Ontology Consortium 2015). The p-value was adjusted using the Benjamini–Hochberg method at FDR < 0.05 for iHS- and ROH-based gene lists and at FDR < 0.1 for Rsb gene lists for GO analysis. Adjusted p-values for the *F_ST_* analysis were set using the Benjamini–Hochberg method (Benjamini and Hochberg 1995) at FDR < 0.05.

### Analysis of environmental variables and genomic variance

The existence of an association between the variants under possible selection and the indigenous environment of the African and Northern European chickens was examined using the latent factor mixed model (lfmm) test (Frichot *et al.* 2013) performed using the R (Gentleman *et al.* 2004) software package LEA (Frichot *et al.* 2013, 2015; Frichot 2015). The software was used to generate a list of candidate loci associated with adaption to the given environmental factors, as well as the number of ancestral populations (K) for the African and Northern European chickens. The lfmm analysis was performed using the 30-yr averages for temperature and precipitation for each of the countries and was performed on each group (African/Northern European) separately. The lfmm analysis was performed several times on each population before taking the median of the merged *z*-scores. The p-values were then adjusted using the computed genomic inflation factor (λ) for the median. The lambda values were chosen to be stringent and were 1 for the African and 1.4 for the Northern European populations. Significance was then based on a corrected FDR value set at *q* ≤ 0.1 using the Benjamini–Hochberg multiple test correction method.

### Ethics statement

Samples were taken from the chicken diversity gene bank at the Friedrich-Loeffler-Institut, which has been established for many years. New samples were collected in Germany in strict accordance to the German Animal Welfare regulations, and relevant notice was given to the authorities of Lower Saxony according to §8 of the German Animal Welfare Act. Breeders were asked for their agreement.

### Data availability

The genotyping data and breed/ecotype identity of the samples analyzed for this study can be obtained upon request from Steffen Weigend (Steffen.Weigend@fli.de).

## Results

### PCA

**Figure 1:**
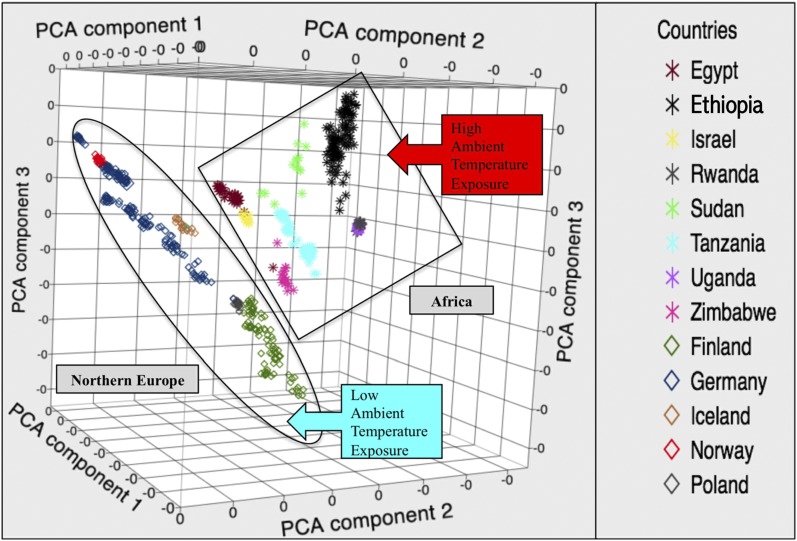
Plot shows that the genotypes and 30-yr temperature profiles for each sampled country differentiated the chickens into their indigenous countries. This differentiation indicates that divergence between the breeds is partly due to environment and that the breeds can be grouped by environment (*i.e.*, higher ambient temperatures and lower ambient temperatures). The oval surrounds the cluster of Northern European countries and the square represents the African countries. PCA, principal component analysis.

PCA of the African and Northern European populations showed that the samples displayed several levels of clustering, which indicated the existence of subpopulations (Figure 1). The first level that showed distinct clusters grouped the samples by country of origin. The next level showed that countries clustered according to continent of origin, with all Northern European birds being grouped together and the Mediterranean and African birds clustering together. The distinct clustering of the PCA demonstrated that temperature profiles, land mass (continent), country, and genotype all separate the data in a similar fashion.

### F_ST_ (case/control)

**Figure 2:**
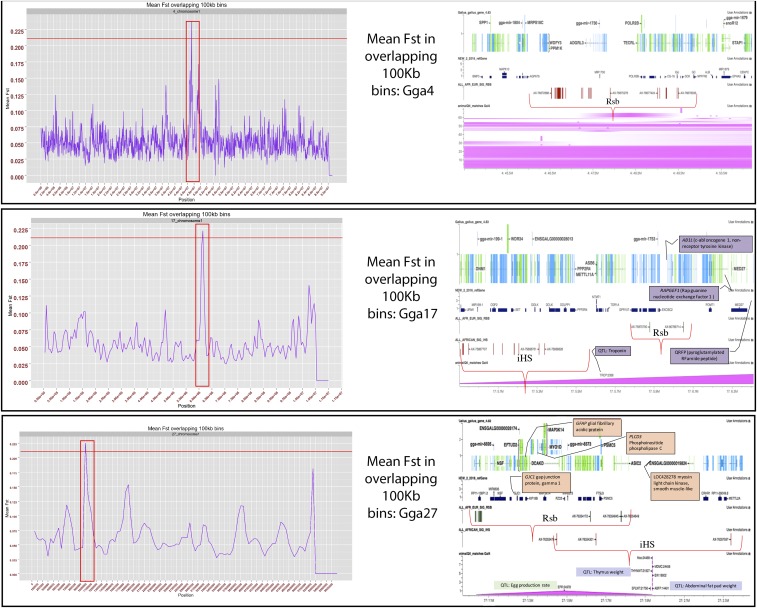
The left side of the figure shows a close-up of the strongest *F_ST_* peaks from the sliding window analysis shown in Figure S1. The right side of the figure shows the genomic architecture under the peaks along with any statistically significant variants from the iHS and Rsb test for selection pressure. The peak on chromosome 4 was the widest area and had the highest *F_ST_* value. The majority of variants that fell within this area from the *F*_ST_ test had higher allele frequency in the Northern European chickens compared to the African chickens. The region under selection on chromosome 17 carries genes involved in redox, Ca^2+^ sequestration, and blood vessel creation. The close-up of the peak on chromosome 17 carries QRFP, a ligand of GPR103 shown to reduce thermogenesis. Chromosome 27 shows possible selection pressure near a recently discovered QTL for body temperature under experimental heat stress conditions meant to simulate climate-induced stress in chickens. Gga, Gallus gallus; iHS, integrated haplotype score; QTL, quantitative trait locus; Rsb, pairs of populations.

The sliding window analysis (Figure S1) indicated that chromosomes 4, 17, and 27 had the strongest *F_ST_* peaks (*F_ST_* > 0.2) (Table 1). The peak on chromosome 4 was from 44 to 51 Mb, contained 131 genes, and was analyzed for GO term enrichment. Cross-referencing of multiple GO enrichment software (FDR ≤ 0.05) showed multiple statistically significant GO terms related to developmental pathways (epidermal growth factor receptor signaling pathway, GO:0007173), calcium signaling (ERBB signaling pathway, GO:0038127; biomineral tissue development, GO:0031214), and immune response (CXCR chemokine receptor binding, GO:0045236; cytokine activity, GO:0005125). The peak on chromosome 17 (Figure 2) covered an area that included 14 genes under the 300 kb peak, possibly differentiated due to selection for the contrasted environments. A total of 19 genes, with the majority being feather keratin-like genes, fell within the peak area on chromosome 27 (Figure 2). This peak overlapped with a region of chromosome 27 under selective pressure based on the iHS results. Genes in this overlap region (1–1.4 Mb) were also considered to be possible candidate genes, and included gap junction protein γ 1(*GJC1*), myosin light chain kinase, smooth muscle-like (LOC428278), glial fibrillary acidic protein (*GFAP*), and phosphoinositide phospholipase C (*PLCD3*) (Table 2).

**Table 1:** GO analysis of mean *F_st_* for 100 kb windows on chromosome 4 (FDR ≤ 0.05)

	Term/Pathway ID
Biological process-GO terms	
Epidermal growth factor receptor signaling pathway	GO:0007173
ERBB signaling pathway	GO:0038127
Biomineral tissue development	GO:0031214
Negative regulation of smooth muscle cell differentiation	GO:0051151
Response to lipid	GO:0033993
Response to nutrient	GO:0007584
Ossification	GO:0001503
Molecular function-GO terms	
CXCR chemokine receptor binding	GO:0045236
Interleukin-8 receptor binding	GO:0005153
Cytokine activity	GO:0005125
Panther database protein classes and KEGG pathway terms	
Toll-like receptor signaling pathway	KEGG:04620
NOD-like receptor signaling pathway	KEGG:04621
InterPro protein domains	
Epidermal growth factor receptor ligand	IPR015497
Serum albumin-like	IPR020858

Summary of GO functions and pathways showing selection pressure on functions related health, adipogenesis, calcium, and inflammatory responses. Many of the GO terms have been shown to be upregulated in cold-stressed chickens (Zhao *et al.* 2014; Napper *et al.* 2015). ID, identifier; GO, gene ontology; KEGG, Kyoto Encyclopedia of Genes and Genomes.

**Table 2:** Candidate genes near peak for sliding mean *F_st_* window for chromosome 27

Gene Name	Function	Possible Relationship to Environmental Stress
Gap junction protein, γ 1 (GJC1)	GO:00015700, vasculogenesis GO:0048738, cardiac muscle tissue development	Smooth muscle contraction, vasodilation (convection)
Myosin light chain kinase, smooth muscle-like: (LOC428278)	GO:0004683, calmodulin-dependent protein kinase activity	Ca^+2^, smooth muscle contraction, vasodilation/constriction
Glial fibrillary acidic protein (GFAP)	GO:1904714, regulation of chaperone-mediated autophagy	Heat shock protein activation
Phosphoinositide phospholipase C (PLCD3)	GO:0006629, lipid metabolic process; GO:0005509, calcium ion binding; GO:0001525 angiogenesis	Lipolysis and vasodilation (convection)

Candidate genes within ±2 Mb of the peak on chromosome 27. The genes in this region overlap with other regions of selection pressure seen in the iHS (integrated haplotype score) and Rsb (pairs of populations) analysis.

### Selection signature analysis: iHS

#### African population:

**Figure 3:**
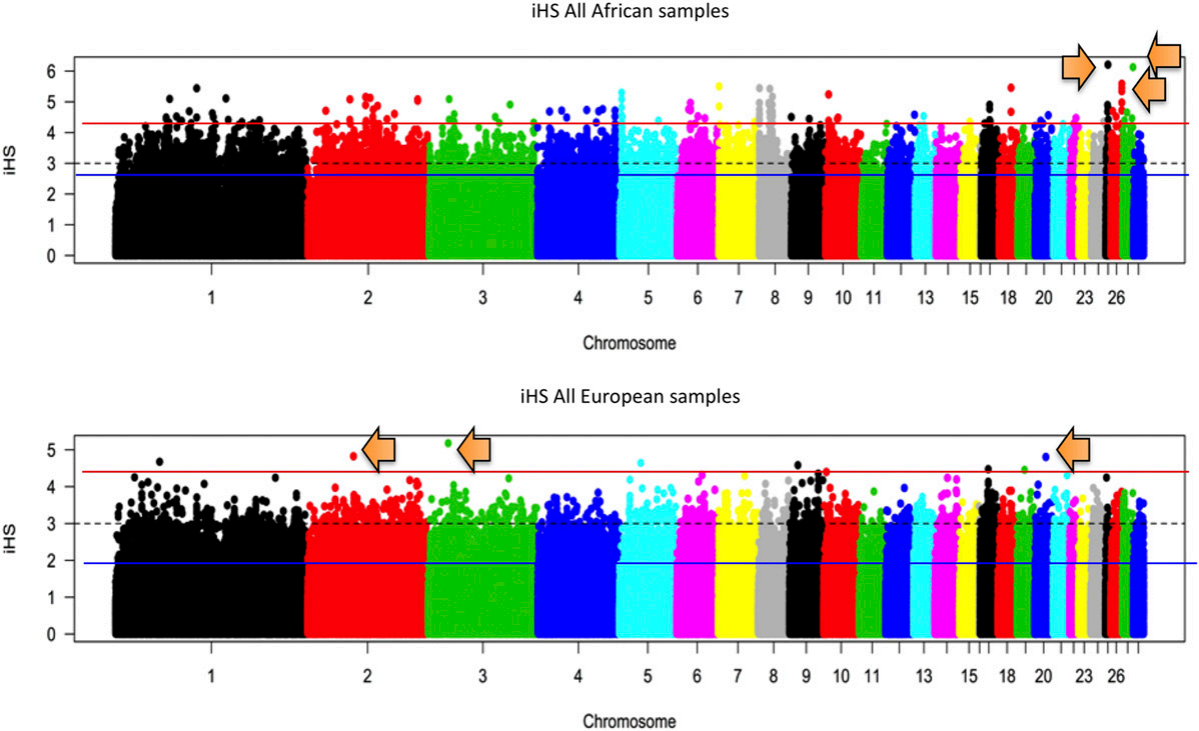
Manhattan plots for the African and Northern European chicken populations. The most extreme iHS peaks were on chromosomes 25, 26, and 27 for the African birds and chromosomes 2, 3, and 20 for Northern European chickens. Plots indicate that selective pressure on both populations is contrasted with selection toward different genomic regions that is likely the result of the difference in habitats. The blue line is the average, black dotted line is cut off at three SD of the mean iHS (= 0), red line shows iHS regions above 3. SD Arrows show highest iHS peaks used for downstream analysis. iHS, integrated haplotype score.

Examination of the iHS SNVs yielded 27 statistically significant SNVs, of which the top three iHS values were on chromosomes 25, 27, and 26, respectively (Figure 3). The genes that had statistically significant variants that clustered in their intronic regions were the highest iHS values considered to be under selective pressure. Chromosome 25 had the highest iHS score and encompassed keratin (LOC769139) and scale keratin-like (LOC769486) genes. The majority of the genes within the ±2 Mb window, which contained the variants on chromosome 25, were related to calcium signaling and feather and claw keratin. The strongest variant under selection on chromosome 27 was within an intron of polymerase I and transcript release factor (*PTRF*) and angiotensin I converting enzyme (peptidyl-dipeptidase A) 1 (*ACE*). Other statistically significant variants fell within introns of thyroid hormone receptor α (*THRA*). Within the ±2 Mb window were the stress related genes hypocretin (orexin) neuropeptide (*HCRT*), heat shock protein 25 (*HSP25*), and DnaJ (Hsp40) homolog, subfamily C, member 7 (*DNAJC7*). The variant under strongest selection on chromosome 26 fell within an intron of BCL2-antagonist/killer 1 (*BAK1*), while other statistically significant variants fell within exon 6 of adenosylhomocysteinase-like 1 (*AHCYL1*) (Table 3). The variant in the coding region of *AHCYL1* caused a deleterious missense mutation that changed a serine (S) to arginine (R) (SIFT score = 0).

**Table 3:** Genes within 2 Mb of statistically significant (≤ 1 × 10^−4^) iHS variants for African and Northern European chickens

	Chr	Location (n/Distance)	Gene	Function/Process
Africa	25	Intron (3)	LOC769139 (keratin)	Intermediate filament (claw, feather, and scale)
	27	Intron (3)	PTRF (Human) (polymerase I and transcript release factor)	Lipid metabolism, insulin-regulated gene expression
	27	Intron (3)	THRA (thyroid hormone receptor, α)	Neuroactive ligand-receptor interaction pathway response to cold (GO:0009409)
	27	Intron (2)	ACE [angiotensin I converting enzyme (peptidyl-dipeptidase A) 1]	Increased vasoconstriction (UniProtKB - Q10751)
				Response to cold (GO:0009409)
	27	Upstream (65 kb)	HCRT [hypocretin (orexin) neuropeptide]	Feeding behavior (GO:0007631)
	27	Upstream (87 kb)	HSP25 (heat shock protein 25)	Response to heat
	27	Upstream (165 kb)	DNAJC7 [DnaJ (Hsp40) homolog, subfamily C, member 7]	Heat shock protein binding
	26	Intron (7)	BAK1 (BCL2-antagonist/killer 1)	Apoptosis (chicken), heat-shock protein chaperone (human)
	26	Exon 6-missense (1)*^a^*	AHCYL1*^a^* (adenosylhomocysteinase-like 1)	Oxidation-reduction process (GO:0055114)
Europe	3	Intron (1)	PTPN14 (protein tyrosine phosphatase, nonreceptor type 14)	Lymphangiogenesis (GO:0001946), Dephosphorylation (GO:0016311)
	3	Downstream (423 kb)	PRKCE (protein kinase C, ɛ)	Positive regulation of insulin secretion (GO:0032024), cellular response to hypoxia (GO:0071456)
	2	Intron (3)	SALL3 [Sal-like 3 (*Drosophila*)]	Limb morphogenesis, smoothened signaling pathway
	2	Downstream (1.6 Mb)	PRL (Prolactin)	Thermal stress in cattle, egg production
	20	Intron (1)	PTPRT (Protein tyrosine phosphatase, receptor type, T)	Dephosphorylation (GO:0016311)
	20	Upstream (1.1 Mb)	DNAJC5 [Dnaj (Hsp40) homolog, subfamily C, member 5 (DNAJC5)]	Negative regulation of neuron apoptotic process (GO:0043524)

Genes in the table were considered to be under strong selection due to the presence of statistically significant variants falling in intragenic regions. The list of genes reads like a list of stress tolerance genes, complete with heat-shock proteins, characterized as key responders to cellular stress events. Chr, chromosome. a, SNV fell within exon.

#### Northern European population:

The chromosomal regions that had the highest significant iHS values for the Northern European chickens were on chromosomes 3, 2, and 20 (Figure 3). The Northern European chickens had less overall statistically significant SNVs and no statistically significant SNVs that clustered within any gene. Because of the limited number of variants under selection in the Northern European populations, significant markers falling on the same chromosome were also viewed within the ±2 Mb window. The highest iHS value on chromosome 3 (single SNV) was located in the intron of protein tyrosine phosphatase, nonreceptor type 14 (*PTPN14*); also under possible selection is protein kinase C ɛ (*PRKCE*). The single SNV of highest significance on chromosome 2 fell within an intron of spalt-like transcription factor 3 (*SALL3*). Another gene near this region of possible importance to the interaction of the Northern European chickens and their environment is the gene prolactin (*PRL*). On chromosome 20, one of the two variants under selective pressure fell within the intron of protein tyrosine phosphatase, receptor type, T (*PTPRT*). Genes of interest within the ±2 Mb window included DnaJ (Hsp40) homolog, subfamily C, member 5 (DNAJC5) and Bcl-2-like protein 1 (*BCL2L1*), along with multiple transglutaminase genes. Chromosome 20 had the highest gene density around significant SNV for the Northern European birds.

#### Selection signature analysis: Rsb:

**Figure 4:**
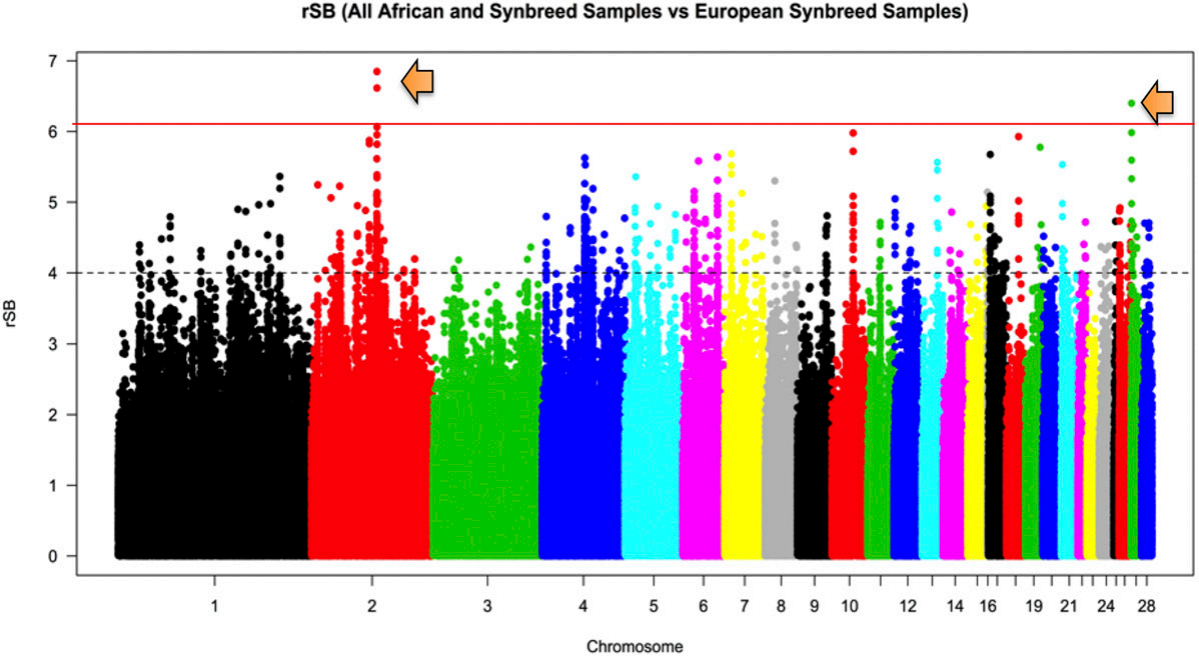
Manhattan plot for the African and Northern European chickens showing the pairwise comparison of selection pressure. Peaks on chromosomes 2 and 27 were analyzed for selection pressure and were shown to be the two chromosomes on which the populations were the most diverged. The data from the *F_ST_* analysis supports the results on chromosome 27 as being under differential selection between the populations. Rsb, pairs of populations. black dotted line is cut off at three SD of the mean iHS (= 0,) red line shows iHS regions above 3 SD.

The results from the pairwise comparison (Rsb) of the African and Northern European chickens identified variants on chromosomes 2 and 27 as having the highest Rsb values (Figure 4). Chromosome 2 had the largest peak and number of statistically significant SNVs between the two groupings. Genes of interest that contained statistically significant variants on chromosome 2 included: IKAROS family zinc finger 1 (Ikaros) (*IKZF1*), von Willebrand factor C domain containing 2 (*VWC2*), TBC1 domain family, member 5 (*TBC1D5*), and interferon regulatory factor 4 (*IRF4*). The significant variants on chromosome 27 fell within the introns of acid-sensing ion channel 2 (*ASIC2*) and in the intron and 3ʹ-UTR of Golgi SNAP receptor complex member 2 (*GOSR2*) (data not shown). Many of the genes highlighted were used to perform a GO analysis to examine which processes and functions showed strong selection pressure differences between the African and Northern European chickens (Table 4).

**Table 4:** GO enrichment analysis of Chr 2 and 27 significant Rsb scores

	GO Enrichment Term (FDR ≤ 0.05)	Term ID	Type
Chr 2	Digestive tract mesoderm development	(GO:0007502)	BP
	Angiogenesis	(GO:0001525)	BP
	Pattern specification process	(GO:0007389)	BP
	Skeletal system development	(GO:0001501)	BP
	Embryo development	(GO:0009790)	BP
	Muscle organ development	(GO:0007517)	BP
	Reproduction	(GO:0000003)	BP
	Nervous system development	(GO:0007399)	BP
	Mucin type O-Glycan biosynthesis	(KEGG:00512)	KE
Chr 27	Canonical Wnt signaling pathway involved in osteoblast differentiation	GO:0044339	BP
	Regulation of systemic arterial blood pressure by aortic arch baroreceptor	GO:0003026	BP
	General adaptation syndrome, behavioral process	GO:0051867	BP
	Frizzled binding	GO:0005109	MF
	NF-κB-inducing kinase activity	GO:0004704	MF
	Melanogenesis	KEGG:04916	KE
	Hedgehog signaling pathway	KEGG:04340	KE
	B cell receptor complex	GO:0019815	CC

GO analysis of the Rsb (pairs of populations) results from chromosome 2 and 27 shows overlap in enriched GO terms seen in the runs of homozygosity and iHS (integrated haplotype score) and *F_ST_* analysis. GO, gene ontology; FDR, false discovery rate; ID, identifier; Chr, chromosome; BP, biological process; KE, Kyoto Encyclopedia of Genes and Genomes; MF, molecular function; CC, cellular component.

### GO analysis of selection signature results (iHS/Rsb)

GO analysis was conducted on the gene lists generated from significant SNVs from the iHS analysis of each grouping and the pairwise comparison of the two. In the African chickens, processes and pathways emerged related to cell death (apoptotic process, GO:0006915), immune function (response to interferon-γ, GO:0034341), and homeostasis (homeostatic process, GO:0042592). The cadherin signaling pathway (P00012) was the only significant pathway in the African chickens (Table S2). In the Northern European chickens, significant molecular functions and biological processes were mostly related to cell adhesion (GO:0007155) and angiogenesis (P00005) (Table S2). Regions of interest used for analysis of overenriched GO terms from the Rsb results for chromosome 2 fell within windows of two separate regions, located from ∼31 to 36 Mb and ∼79 to 83.7 Mb. The window on chromosome 27 was from ∼1 to 3 Mb. Data from the Rsb analysis was statistically significant (FDR < 0.1) for biological processes involved in digestive tract mesoderm development (GO:0007502), angiogenesis (GO:0001525), pattern specification process (GO:0007389), skeletal system development (GO:0001501), and mucin type O-Glycan biosynthesis (KEGG:00512) on chromosome 2. Overenriched pathways and processes on chromosome 27 included frizzled binding (GO:0005109), NF-κB-inducing kinase activity (GO:0004704), melanogenesis (KEGG:04916), and regulation of systemic arterial blood pressure by aortic arch baroreceptor (GO:0003026) (Table S2).

### ROH (landscape consensus ROH)

Analysis of all (*N* = 634) chickens in the study yielded a total of 4167 overlapping pools of ROH. Analysis of the overlapping consensus regions revealed that the Northern European chickens harbored 22 pools of LROH that were unique to the Northern European birds. The unique ROH were located on chromosomes 1, 2, and 4. Chromosome 2 had the largest number of ROH pools and individuals. Genes within the consensus ROH were used to carry out GO and pathway analysis for chromosomes 1 and 2 as there were no genes in the region on chromosome 4. The single region on chromosome 1 contained only eight genes, of which calcium channel, voltage-dependent, L type, α 1C subunit (*CACNA1C*), interleukin 17 receptor A (*IL17RA*), and BCL2-like 13 (apoptosis facilitator) (*BCL2L13*) appeared to be biologically relevant. The statistically significant processes related to the consensus LROH (Table 5) for chromosomes 1 and 2 included multiple hits related to glycolysis, neural signaling, and protein inhibition and recycling. There were known QTL for body morphological traits and intramuscular fat that were almost completely within ROH on chromosome 2 (Figure S1 and S2).

**Table 5:** GO enrichment analysis of chromosome 1 and 2 landscape ROH

GO enrichment term (FDR ≤ 0.05)	Term ID	Type
Chromosome 1		
Galactose metabolism	KEGG:00052	Pathway
Pentose and glucuronate interconversions	KEGG:00040	Pathway
Fructose and mannose metabolism	KEGG:00051	Pathway
γ-aminobutyric acid: sodium symporter activity	GO:0005332	MF
γ-aminobutyric acid transmembrane transporter activity	GO:0015185	MF
Sodium: amino acid symporter activity	GO:0005283	MF
Cation: amino acid symporter activity	GO:0005416	MF
Aldo/keto reductase family	PF00248	Pfam
NADP-dependent oxidoreductase domain	IPR023210	InterPro
Chromosome 2		
Negative regulation of signal transduction in absence of ligand	GO:1901099	BP
Negative regulation of extrinsic apoptotic signaling pathway in absence of ligand	GO:2001240	BP
Protein localization to cell junction	GO:1902414	BP
Positive regulation of lipopolysaccharide-mediated signaling pathway	GO:0031666	BP
Serine protease inhibitor	PC00204	Protein
Serine-type endopeptidase inhibitor activity	GO:0004867	MF
Peptidase inhibitor activity	GO:0030414	MF

GO analysis of overrepresented genes in from the landscape run of homozygosity (ROH) analysis shows that the Northern European chickens favor mechanisms involved in inhibition of protein digestion, apoptosis, and possibly amino acid recycling. GO, gene ontology; FDR, false discovery rate; ID, identifier; KEGG, Kyoto Encyclopedia of Genes and Genomes; MF, molecular function; BP, biological process.

### Environmental factor association analysis (lfmm)

The lfmm analysis of the African and Northern European chicken populations returned results on the diversity of the population structures (K) and listed statistically significant SNV loci associated with possible adaptation to the environmental factors of temperature and precipitation. The African chicken population exhibited a heavily admixed population structure with a *K* = 9 (out of 23 breeds/ecotypes from sampling) (Figure S6), and had candidate loci lists that totaled 2489 for temperature and 1961 for precipitation. The Northern European chicken population was only slightly stratified with a *K* = 19 (out of 22 breeds from sampling) (Figure S6) and produced a candidate loci list of 9738 for temperature and 7676 for precipitation. Some of the candidate loci discovered to be associated with selective pressure to adapt to the environment overlapped with genomic positions from the iHS, *F_ST_*, Rsb, and ROH analyses. In the African chicken population, this included chromosomes 25, 26, and 27 from the iHS analysis (Table S2). In the Northern European chickens, candidate loci overlapped with iHS regions on chromosomes 2, 3, and 20 (Table S2). The lfmm analysis supported the previous results for candidate loci near genes of interest under selective pressure.

## Discussion

The stochastic nature of the connection between genotype, stress, environment, selective pressure, and adaptive phenotype makes it difficult to attribute changes in any of these phenomena to any single stressor or genetic phenomenon. Although, it is possible that different populations or breeds may converge around an adaptive advantage through the process of random drift, it is unlikely that random drift would leave identifiable “footprints” across multiple breeds due to its randomness. To identify and examine the “footprints” and the effects that contrasted environments have on the genomic architecture, historical temperature profiles of the countries were used as proxies for environment to account for potential differences in thermal stress. By using thermal stress as proxy for environmental stress, we can examine multiple issues related to environmental stress in livestock and view the data by genomic and physiological processes related to environmental stress tolerance in general, and thermal stress tolerance specifically. The biological pathways and network functions based upon the genomic variation of the chickens that address these may offer the information needed to select for environmental stress tolerance of multiple climates (Seebacher 2009; Walter and Seebacher 2009; Valero *et al.* 2014). Despite this, many of the processes and genes found to be under selection are part of a general stress (Zakrzewska *et al.* 2011) response meant to provide adaptability to multiple stressors in the animal’s environment, which may or may not be quantifiable or captured by temperature as a proxy (Clarke 2003; Canario *et al.* 2013; Newman *et al.* 2013; Zhao *et al.* 2014; Napper *et al.* 2015; Nguyen *et al.* 2015; H. Y. Sun *et al.* 2015; L. Sun *et al.* 2015; Van Goor *et al.* 2015). PCA was able to show us that the samples could be split into two distinct groupings based upon divergence in the populations by both genotype and environment. The overlap between these two categories seems to indicate that each group of chickens (African/Northern European) has evolved genetic functions related to survival of their specific, indigenous environments that should present as distinct differences in their genomic architecture. The PCA results allowed the analysis to be performed as a case/control study, with temperature as a proxy phenotype, to allow the results to be viewed as genomic regions under possible selection for environmental stress tolerance differing by population and related to each population’s indigenous environment. The examination of the African and Northern European chicken populations utilized multiple methods that showed overlap in the genomic regions carrying selection signals. The overlap between methods presented chromosomes 2 and 27 as being highly differentiated between the two groups, and under high selective pressure within each group (Northern Europe and Africa), respectively.

### Selection signatures unique to the divergence of the African chickens

The results on chromosome 27 may be the most biologically relevant results from the iHS analysis, due to their overlap with results from the *F_ST_* and Rsb analyses that indicate there is selective pressure in the region of ∼1–4 Mb of chromosome 27 within the African (high ambient temperature) populations. Within this area is the gene *THRA*, which has homologs with functions related to environmental stress (response to cold GO:0009409) and muscle function in the heart (regulation of heart contraction GO:0008016). The area of chromosome 27 overlapped by the *F_ST_*, iHS, and Rsb results also holds the genes *HSP25* and *DNAJC7*, both which are directly related to stress tolerance responses (Jenuth 2000; UniProt Consortium 2015). The presence of selective pressure where these genes are located could be indicative of a region involved in environmental stress responses in chicken. The gene *HSP25* has been shown to be upregulated in indigenous Taiwanese chickens (hot climate) (Wang *et al.* 2015) under acute heat stress, which supports the idea of selection toward this gene in warmer habitats. The gene *ASIC2*, under selective pressure on chromosome 27, also supports selection toward conductance for heat tolerance, but also helps regulate pH balance, which could indicate homeostatic functioning. The strongest evidence of selective pressure on chromosome 27 being related to thermal tolerance, by more than just proxy, is demonstrated by the selection around the *ACE* gene involved in vasculogenesis (Jenuth 2000). Van Goor *et al.* (2015) reported that both the *ACE* gene and a previously unreported QTL for body temperature (BT28-20) under heat stress mapped to the same region(s) identified in the current study as being under selective pressure in the African population. The QTL region found by Van Goor *et al.* (2015) on chromosome 27, and the areas under selection in the African chickens, overlap around several possible candidate genes in addition to *ACE*. The results from both studies appear to point to a region of chromosome 27 that is involved in heat tolerance, and this region may have been selected for a thermal advantage in Africa’s ancient chickens and persisted through the continued domestication and improvement of African poultry breeds and ecotypes. The lack of selective pressure in this region among the Northern European chickens that were analyzed may suggest that the region is tied to some functional advantage that is useful for hotter environments. Analysis of the GO enrichment for chromosome 27 based on the iHS and Rsb results also indicated a heat tolerance region located in the vicinity of ∼1–3 Mb on chromosome 27. The terms homeostatic process (GO:0042592), regulation of systemic arterial blood pressure by aortic arch baroreceptor (GO:0003026), frizzled binding (GO:0005109), and melanogenesis (KEGG:04916) were all overenriched for the region under selection on chromosome 27 (Table S2). Studies have shown that genes involved in these processes are differentially regulated under experimentally generated heat stress (Gerken *et al.* 2006; Li *et al.* 2014; H. Y. Sun *et al.* 2015; L. Sun *et al.* 2015).

### Selection signatures unique to the divergence of the Northern European chickens

The regions under the strongest selection pressure in the Northern European birds were located on chromosomes 3, 2, and 20 (Table 3). Chromosomes 3 and 20 both have genes that function in dephosphorylation and thermal stress and may indicate selection toward thermogenesis through energy uncoupling as a means of generating body heat in order to achieve cold tolerance in Northern European chickens (Seebacher 2009; Newman *et al.* 2013; Zhao *et al.* 2014; Napper *et al.* 2015). Another gene of biological interest on chromosome 3 from the Northern European iHS analysis is *PRKCE*. The gene has functions related to insulin secretion and response to low oxygen environments in chickens (Jenuth 2000; UniProt Consortium 2015; Herrero *et al.* 2016; Kersey *et al.* 2016). In humans, *PRKCE* is considered a stress response gene involved in cardiac tissue (Barnett *et al.* 2007); however, its impact is less defined in chickens. The selection signatures in the Northern European birds are possibly related to stress and/or some advantage in sugar oxidation that increases thermogenesis. Selection toward *PRKCE* also supports the hypotheses from previous work that suggest that kinases, such as *PRKCA*, were involved in the response to the environment in poultry. Studies have demonstrated that both *PRKCA* and *PRKCE* can have opposite effects on NADPH usage (F. Chen *et al.* 2014), a process that seems to be under unique selection in the Northern European chickens and echoed in the LROH GO enrichment results for chromosome 1 (Table 5). However, the major overlap in genomic regions that showed evidence of selection signatures identified by multiple methods (iHS, Rsb, and LROH) occurred on chromosome 2. The variants under selective pressure on chromosome 2 from the iHS (Table 3) analysis intersect with the LROH (Figure S3 and Figure S4) and seem to signify selective pressure around the *PRL* gene. In relation to environment, *PRL* has been shown to have variants that can cause tolerance phenotypes to heat or cold stress in cattle that affect hair morphology (Littlejohn *et al.* 2014). Likewise, variation in the *PRL* gene in turkeys and the *PRLR* receptor in chickens has been shown to affect feathering (Smyth 1954; Elferink *et al.* 2008) and may represent tolerance to the environment involving feather structure for improved evaporation, insulation, or filtration in the Northern European chickens. The terms negative regulation of ossification and negative regulation of nitric oxide mediated signal transduction (GO:0010751) are associated phenotypes with *PRL* that may putatively be involved in increased skeletal muscle development and skeletogenesis. Additionally, these GO terms have been observed in studies of cold tolerant birds (Walter and Seebacher 2009; Newman *et al.* 2013). Nitric oxide is capable of stimulating perfusive heat loss (Seebacher 2009), which Northern European chickens may have been selected against as an evolutionary adaptation. Also on chromosome 2 is evidence of population-specific selection on the *SALL3* gene alleles based on the iHS analysis and on *IKZF1*, *TBC1D5*, and *IRF4* alleles from the Rsb analysis. These genes are involved in processes that can be seen in experimental studies related to hypothermia and stress (Muhammad *et al.* 2008; Pinard-van der Laan *et al.* 2009; Seaman *et al.* 2009; Cheviron *et al.* 2014; Zhao *et al.* 2014). The variants annotated to these genes also fell within a selective sweep previously described in Qanbari *et al.* (2012), supplying more evidence of the existence of selection toward this area in natural populations.

When compared to the African population, the results of GO analysis of the LROH, iHS, and Rsb seem to indicate possible selection for metabolic processes that employ ion channel transport driven by sugar oxidation and NADPH^+^ as an alternative means of energy metabolism in response to the environment of Northern Europe (Table 3 and Table 5). Additionally, unlike the African iHS data (Figure S5), there proved to be no identifiable signaling networks in the Northern European birds based on the genes annotated to the iHS. It is possible that the Northern European chickens are more dependent on the pentose phosphate pathway than the glycolysis pathway. It is also possible that the selective pressure differences in energy metabolism pathways seen in the Northern European birds have been influenced over time by different nutrient availability. Taken together, the region on chromosome 2 could be related to energy metabolism, inhibition of protein digestion, and fatty acid oxidation (X. Y. Chen *et al.* 2014b; Nguyen *et al.* 2015) as naturally occurring tolerance mechanisms in indigenous Northern European chickens. Considering that these results are unique to birds from the Northern European environment, the discovery of these LROH may point to a selection signature for adaptation to temperate or cold conditions.

### Conclusions

The data from the *F_ST_*, LROH, iHS, and Rsb analyses all support the presence of strong selection pressure on regions of chromosomes 2 and 27. The genes and processes under selective pressure on chromosome 27 are unique to the African populations as there were no significant iHS scores for any chromosome 27 variants in the Northern European chicken populations sampled. The same proved true for chromosome 2 within the African populations. The Northern European chickens appear to be under less selective pressure than the African population, as evidenced by fewer variants reaching significance. The difference in the number of variants under selection could possibly reflect a less diverse or less harsh environment being encountered by the Northern European birds due to their status as heritage birds. The differences in variant numbers may also possibly reflect less change in one environment *vs.* the other (high ambient *vs.* low ambient temperature) over time. It is possible that, when viewed as being entirely subject to their environment, the Northern European birds may have had some relaxation in environmental pressures of thermal stress under modern husbandry; however, the African chickens are mostly village chickens in a rural, scavenger/forage type of farming system allowing for more environmental exposure over more diverse environments (*i.e.*, desert, mountain, jungle, or basin). The study supports the suggestion from experimental studies of chickens under heat stress that chromosome 27 is related to tolerance and survival under stressful environmental conditions. This result proved applicable in both experimental and natural populations encountering stress from high ambient temperatures. Additionally, the study also gives novel insights into unique genomic regions on chromosome 2 in the Northern European chickens that are possibly related to development and environment. Overall, the results indicate that environmental differences may have a part in selection for some portion of the genomic divergence seen in development and metabolic processes of these very distinct populations.

## Supplementary Material

Supplemental material is available online at www.g3journal.org/lookup/suppl/doi:10.1534/g3.117.041228/-/DC1.
