# Supplementary figures and images for: Genomic Comparison of Indigenous African and Northern European Chickens Reveals Putative Mechanisms of Stress Tolerance Related to Environmental Selection Pressure
Source: G3 (Bethesda). 2017 Mar 22;7(5):1525–37. doi: 10.1534/g3.117.041228 (PMC5427493; doi:10.1534/g3.117.041228)

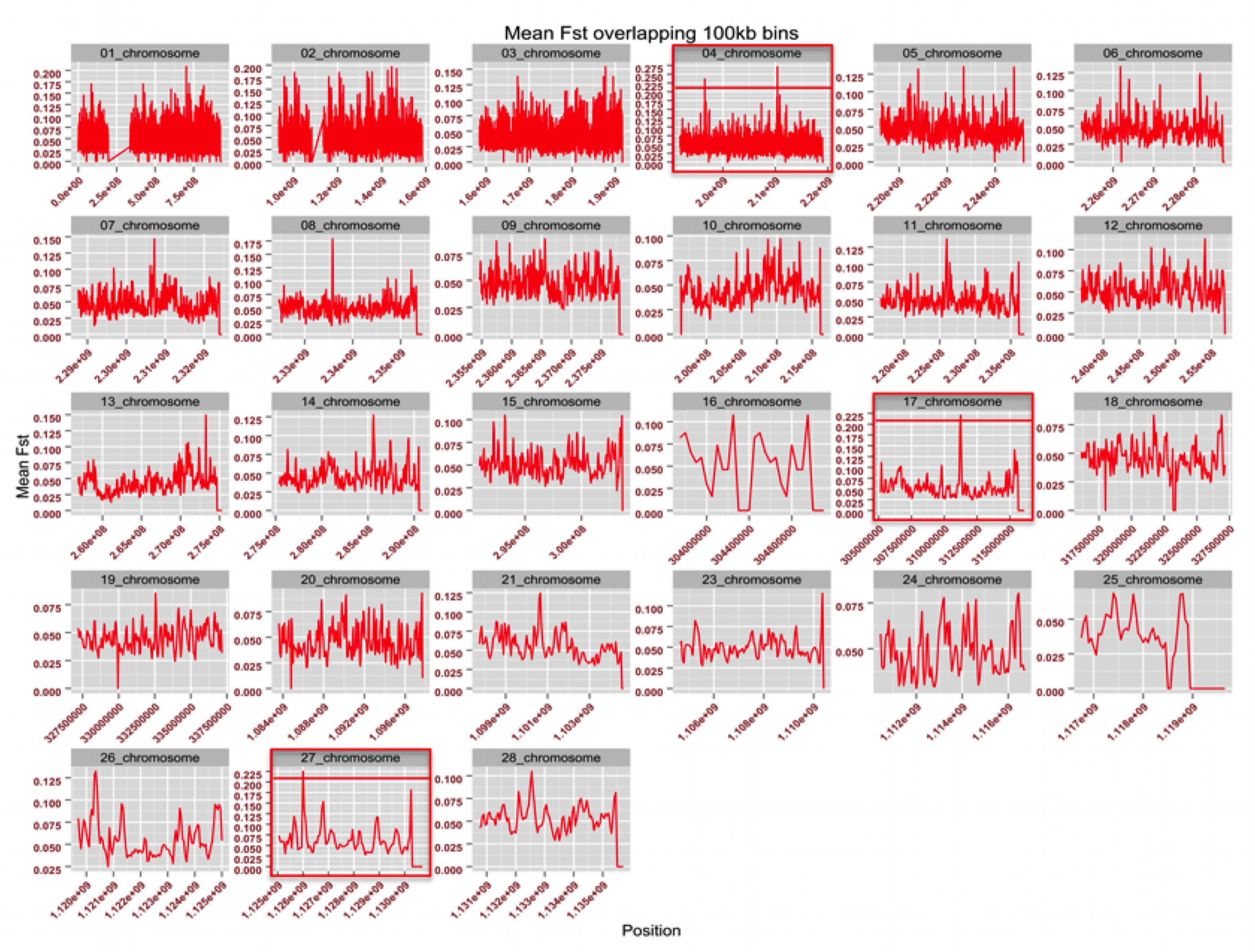

Supplement: Supplementary file 1 [file 1525FigureS1.tif]

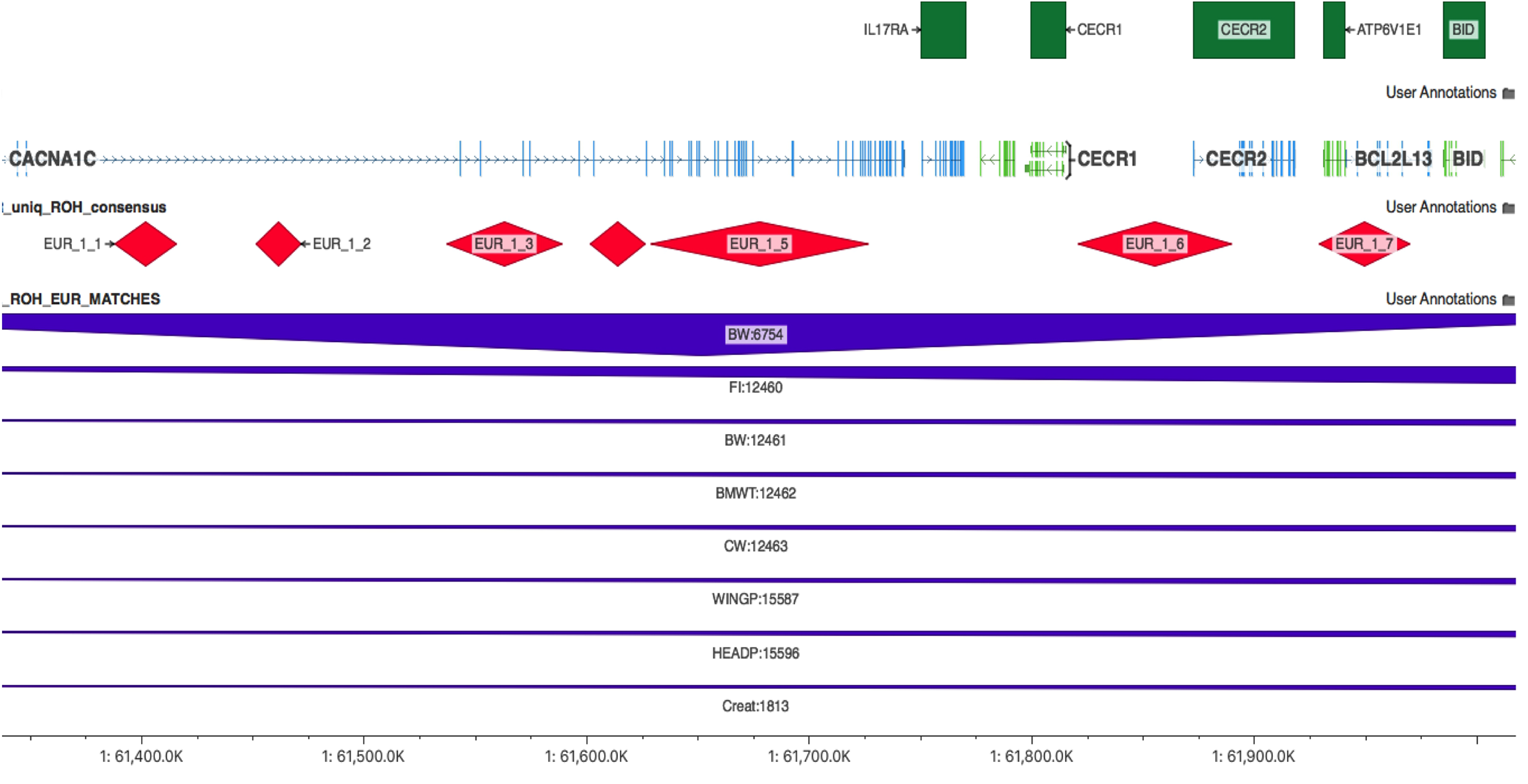

Supplement: Supplementary file 2 [file 1525FigureS2.tif]

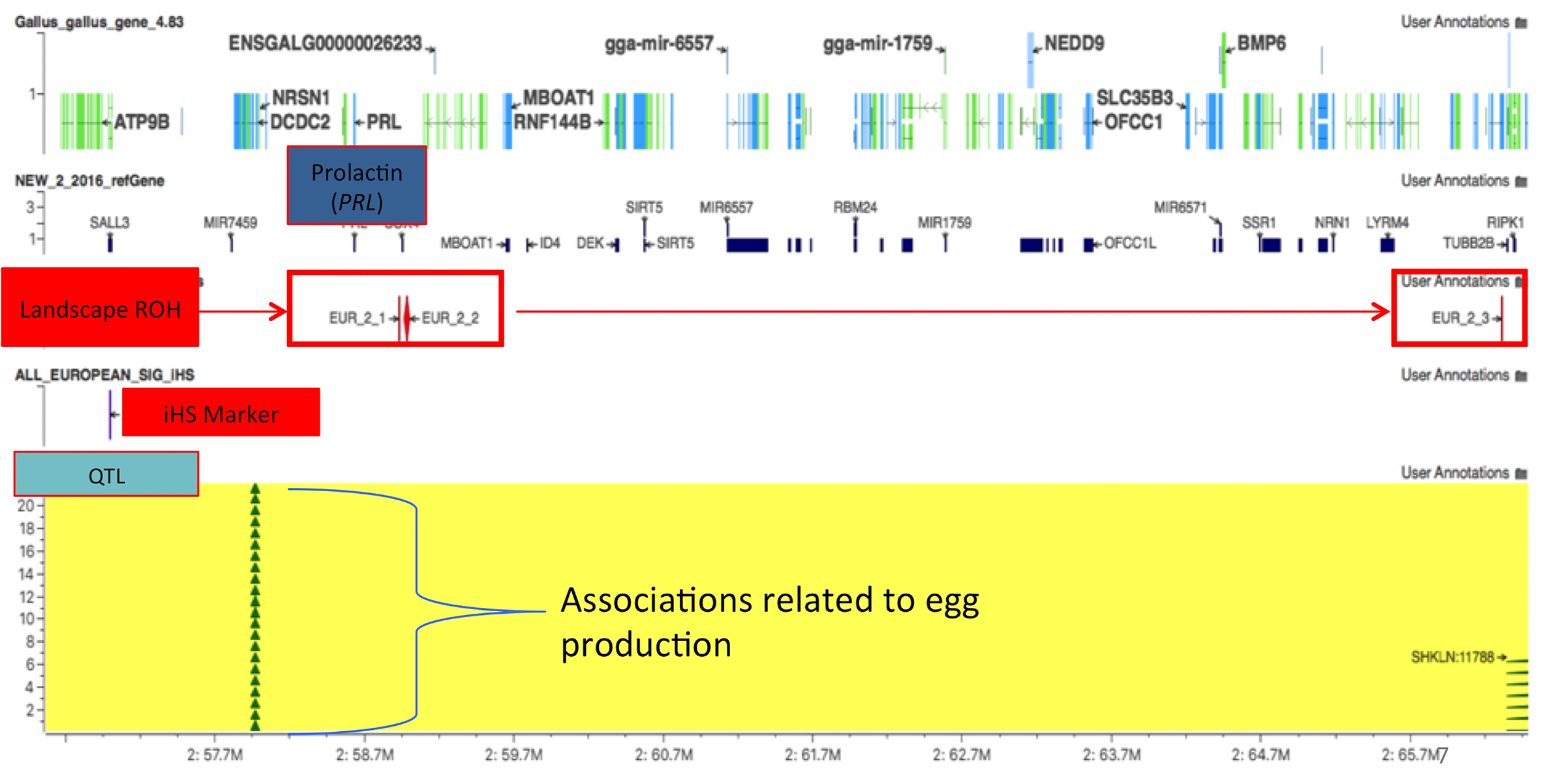

Supplement: Supplementary file 3 [file 1525FigureS3.tif]

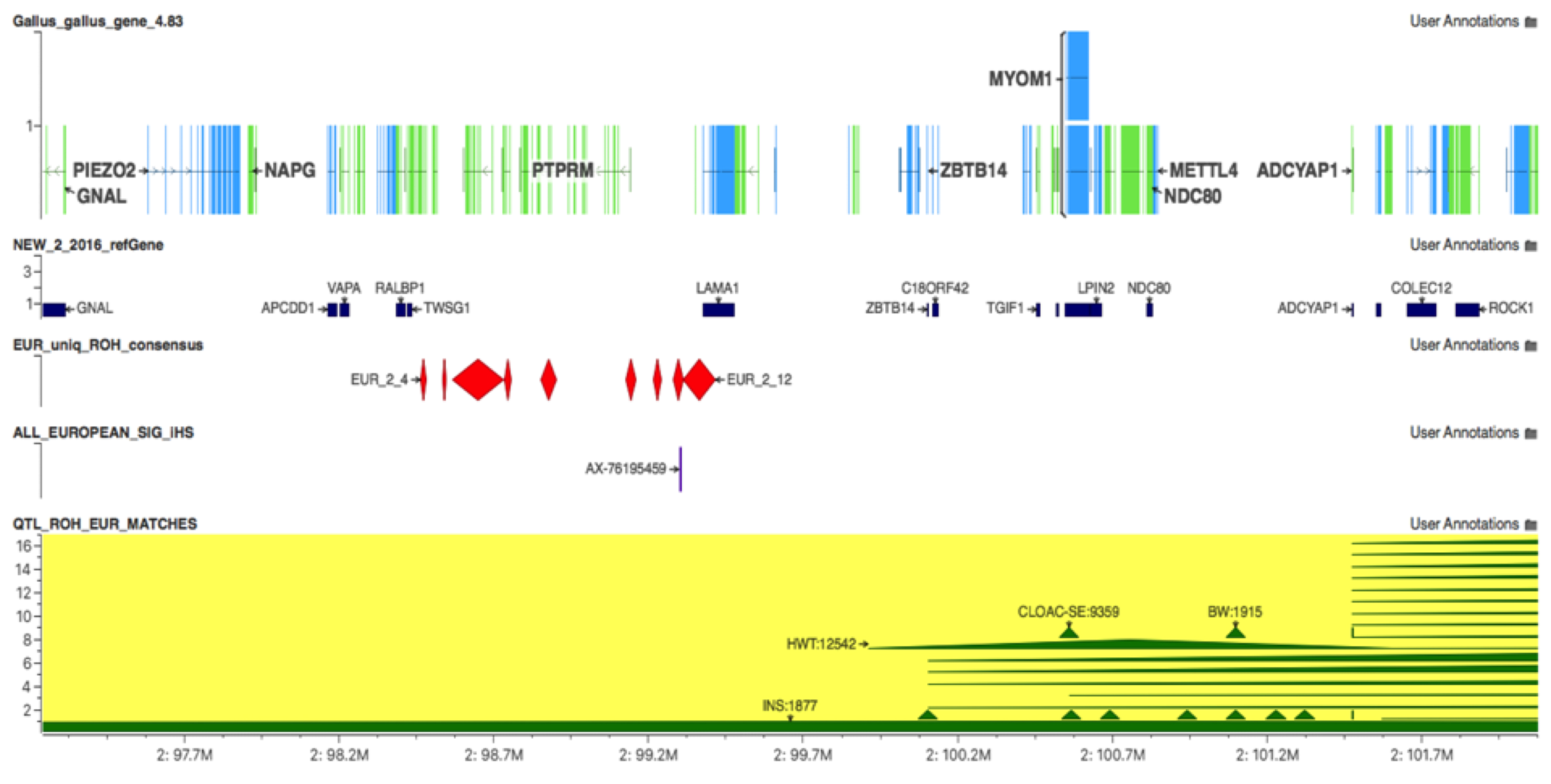

Supplement: Supplementary file 4 [file 1525FigureS4.tif]

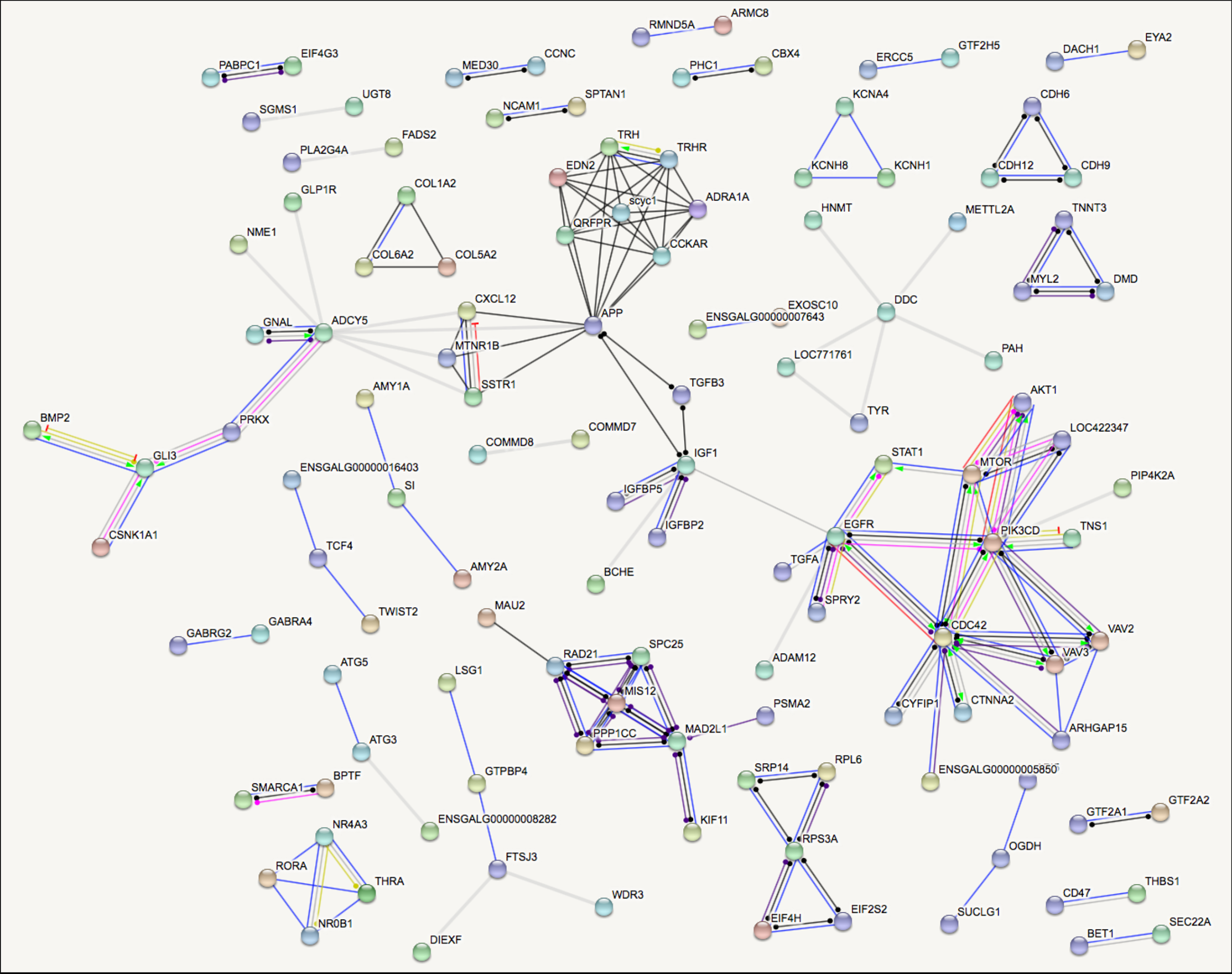

Supplement: Supplementary file 5 [file 1525FigureS5.tif]

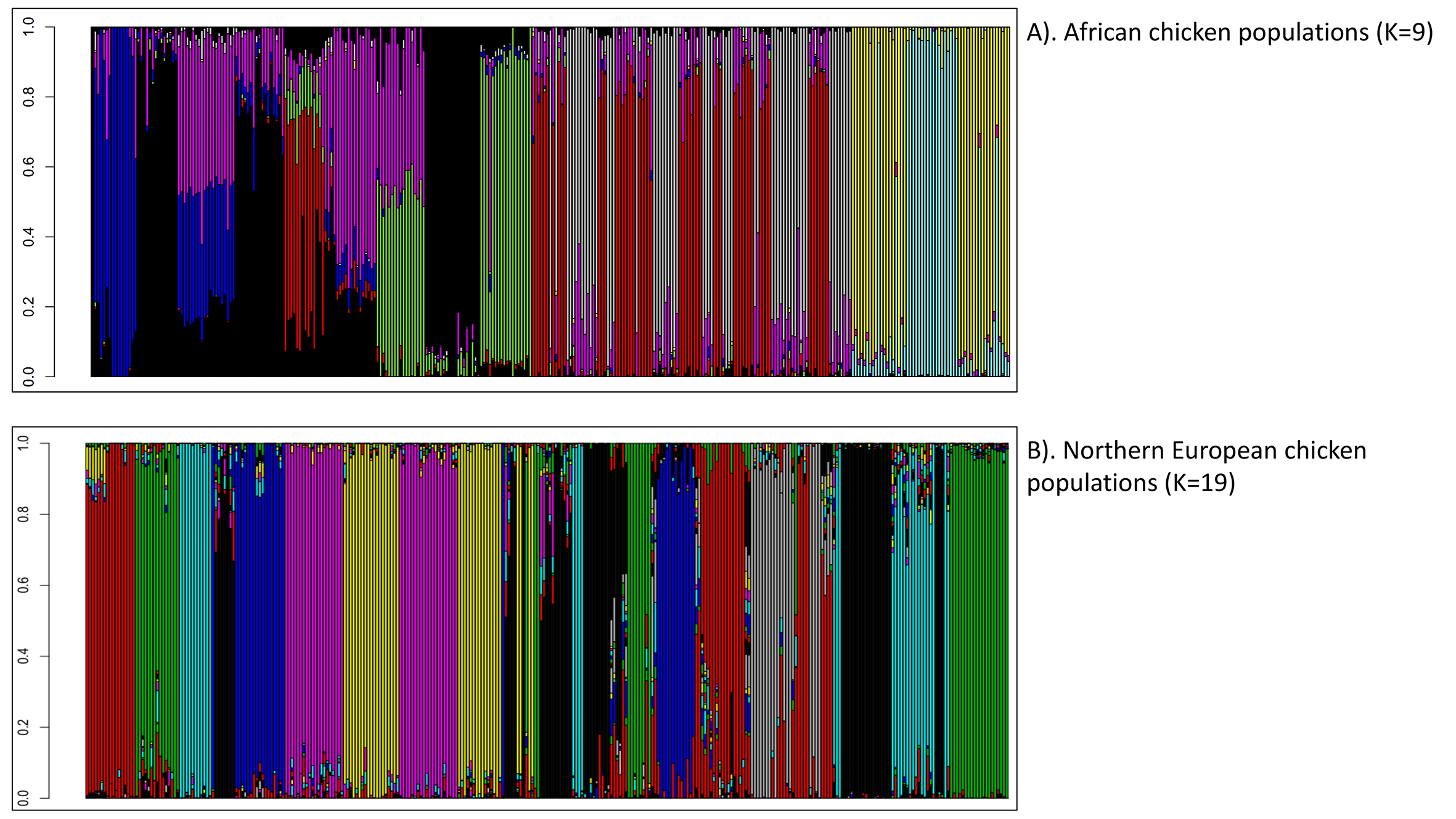

Supplement: Supplementary file 6 [file 1525FigureS6.tif]
