# Supplementary material for: Genomic Comparison of Indigenous African and Northern European Chickens Reveals Putative Mechanisms of Stress Tolerance Related to Environmental Selection Pressure
Source: G3 (Bethesda). 2017 Mar 22;7(5):1525–37. doi: 10.1534/g3.117.041228 (PMC5427493; doi:10.1534/g3.117.041228)
Supplement: Supplementary file 7 [file 1525TableS1.docx]

Supplementary table A. Temperature and precipitation profile by breed and country

| Land Mass | Country | Breed | Annual temperature means (C°) (NOAA, World bank data 1960-1990) | Annual temperature sd (C°) (NOAA, World bank data 1960-1990) | Evolutionary ambient temperature exposure (high=case, low=control) |
| --- | --- | --- | --- | --- | --- |
| Africa | Egypt | Dandarawi | 22.3 | 5.96 | High |
|  | Egypt | Fayoumi | 22.3 | 5.96 | High |
|  | Ethiopia | HA | 22.9 | 1.08 | High |
|  | Ethiopia | HB | 22.9 | 1.08 | High |
|  | Ethiopia | JA | 22.9 | 1.08 | High |
|  | Ethiopia | JB | 22.9 | 1.08 | High |
|  | Israel | Bedouin | 19.5 | 5.56 | High |
|  | Rwanda | Local ecotype | 18.7 | 0.45 | High |
|  | Sudan | Bare Neck | 26.7 | 2.88 | High |
|  | Sudan | Betwil | 26.7 | 2.88 | High |
|  | Sudan | large beladi | 26.7 | 2.88 | High |
|  | Tanzania | Chingwekwe | 22.4 | 1.23 | High |
|  | Tanzania | Kuchi | 22.4 | 1.23 | High |
|  | Tanzania | Morogoro Medium | 22.4 | 1.23 | High |
|  | Tanzania | Pemba | 22.4 | 1.23 | High |
|  | Tanzania | Unguja | 22.4 | 1.23 | High |
|  | Uganda | Local ecotype | 22.5 | 0.83 | High |
|  | Uganda | Kuroiler | 22.5 | 0.83 | High |
|  | Zimbabwe | Ecozone1 | 21.3 | 3.26 | High |
|  | Zimbabwe | Ecozone2 | 21.3 | 3.26 | High |
|  | Zimbabwe | Ecozone3 | 21.3 | 3.26 | High |
|  | Zimbabwe | Ecozone4 | 21.3 | 3.26 | High |
|  | Zimbabwe | Ecozone5 | 21.3 | 3.26 | High |
| Northern Europe | Finland | ALH | 1.23 | 9.76 | Low |
|  | Finland | HÃM | 1.23 | 9.76 | Low |
|  | Finland | HOR | 1.23 | 9.76 | Low |
|  | Finland | IIT | 1.23 | 9.76 | Low |
|  | Finland | ILM | 1.23 | 9.76 | Low |
|  | Finland | JUJ | 1.23 | 9.76 | Low |
|  | Finland | KIU | 1.23 | 9.76 | Low |
|  | Finland | LUU | 1.23 | 9.76 | Low |
|  | Finland | PII | 1.23 | 9.76 | Low |
|  | Finland | SAV | 1.23 | 9.76 | Low |
|  | Finland | TYR | 1.23 | 9.76 | Low |
|  | Germany | Brakel | 8.36 | 6.54 | Low |
|  | Germany | Deutsche Sperber | 8.36 | 6.54 | Low |
|  | Germany | Hamburger | 8.36 | 6.54 | Low |
|  | Germany | Krueper | 8.36 | 6.54 | Low |
|  | Germany | Lakenfelder | 8.36 | 6.54 | Low |
|  | Germany | Ostfriesische Moewen | 8.36 | 6.54 | Low |
|  | Germany | Rheinlaender | 8.36 | 6.54 | Low |
|  | Germany | Westfaelische Totleger | 8.36 | 6.54 | Low |
|  | Iceland | Icelandic Landrace | 1.75 | 4.44 | Low |
|  | Norway | Jaerhoens | 0.88 | 7.53 | Low |
|  | Poland | Green-legged Partridge | 7.75 | 7.46 | Low |
|  |  |  |  |  |  |

Table lists the 30-year averages for temperature and precipitation as averaged across an entire country as reported by the National Oceanic and Atmospheric Administration (NOAA).
