# Supplementary material for: Genomic Comparison of Indigenous African and Northern European Chickens Reveals Putative Mechanisms of Stress Tolerance Related to Environmental Selection Pressure
Source: G3 (Bethesda). 2017 Mar 22;7(5):1525–37. doi: 10.1534/g3.117.041228 (PMC5427493; doi:10.1534/g3.117.041228)
Supplement: Supplementary file 8 [file 1525TableS2.docx]

| Population | Chromosome | Gene | # of SNVs (+/- 1Mb) | |
| --- | --- | --- | --- | --- |
| African | 26 | *BAK1* | | 13 |
|  | 26 | *AHCYL1* | | 26 |
|  | 27 | *ACE* | | 17 |
|  | 27 | *GJC1* | | 36 |
|  | 27 | *GFAP* | | 36 |
|  | 27 | *THRA* | | 5 |
|  | 27 | Multiple LOC (Feather keratin/keratin-like genes) | | 7 |
| Northern European | 2 | *SALL3* | | 108 |
|  | 2 | *PRL* | | 119 |
|  | 3 | *PTPN14* | | 20 |
|  | 3 | *PRKCE* | | 19 |
|  | 20 | *PTPRT* | | 64 |

Supplementary table B. Candidate genes from the lfmm analysis
